# The protective role of microRNA-21 against coxsackievirus B3 infection through targeting the MAP2K3/P38 MAPK signaling pathway

**DOI:** 10.1186/s12967-019-2077-y

**Published:** 2019-10-04

**Authors:** Feng He, Zonghui Xiao, Hailan Yao, Sen Li, Miao Feng, Wei Wang, Zhewei Liu, Zhuo Liu, Jianxin Wu

**Affiliations:** 1 Department of Biochemistry & Immunology, Capital Institute of Pediatrics-Peking University Teaching Hospital, YaBao Road 2, Beijing, 100020 China; 2 Department of Biochemistry & Immunology, Capital Institute of Pediatrics, YaBao Road 2, Beijing, 100020 China

**Keywords:** CVB3, miRNA-21, P38 MAPK, Viral release

## Abstract

**Background:**

The P38 mitogen-activated protein kinase (MAPK) pathway plays an essential role in CVB3-induced diseases. We previously demonstrated microRNA-21 has potential inhibitory effect on the MAP2K3 which locates upstream of P38 MAPK and was upregulated in mouse hearts upon CVB3 infection. However, the effect and underlying mechanism of miRNA-21 on CVB3 infection remain unclear.

**Methods:**

We detected continuous changes of cellular miRNA-21 and P38 MAPK proteins expression profiling post CVB3 infection in vitro within 12 h. P38 MAPK signaling was inhibited by the specific inhibitor, small interfering RNA and miRNA-21 mimic in vitro, CVB3 replication, cell apoptosis rate and proliferation were detected. Viral load in the mice heart, cardiomyocyte apoptosis rate and histological of the heart were also detected in the mice model of viral myocarditis pretreated with miRNA-21-lentivirus.

**Results:**

We observed significant upregulation of miRNA-21 expression followed by suppression of the MAP2K3/P38 MAPK signaling in CVB3-infected Hela cells. The inactivation of the MAP2K3/P38 MAPK signaling by P38 MAPK specific inhibitor, small interfering RNA against MAP2K3, or miRNA-21 overexpression significantly inhibited viral progeny release from CVB3-infected cells. Mechanistically, when compared with control miRNA, miRNA-21 showed no effect on capsid protein VP1 expression and viral load within host cells, while significantly reversing CVB3-induced caspase-3 activation and cell apoptosis rate, further promoting proliferation of infected cells, which indicates the inhibitory effect of miRNA-21 on CVB3 progeny release. In the in vivo study, when compared with control miRNA, miRNA-21 pretreatment remarkably inactivated the MAP2K3/P38 MAPK signaling in mice and protected them against CVB3 infection as evidenced by significantly alleviated cell apoptosis rate, reduced viral titers, necrosis in the heart as well as by remarkably prolonged survival time.

**Conclusions:**

miRNA-21 were reverse correlated with P38 MAPK activation post CVB3 infection, miRNA-21 overexpression significantly inhibited viral progeny release and decreased myocytes apoptosis rate in vitro and in vivo, suggesting that miRNA-21 may serve as a potential therapeutic agent against CVB3 infection through targeting the MAP2K3/P38 MAPK signaling.

## Background

Coxsackievirus B3 (CVB3) is a common and important pathogen of viral myocarditis, pancreatitis, and aseptic meningitis in young children and infants. CVB3 infection may lead to acute heart failure and sudden death due to direct cytopathic effects induced by viral replication in the early phase of infection [1, 2]. Virus elimination from vital organs is considered as a key therapeutic strategy to treat CVB3-associated diseases. However, currently available drugs for the treatment of CVB3 infection are broad-spectrum antiviral drugs with limited efficacy [3–5]. Therefore, understanding of the mechanisms involved in CVB3 infection may provide new clues for developing novel therapeutic strategies against CVB3-induced diseases.

Mitogen-activated protein kinases (MAPKs) are a family of protein kinases involved in converting extracellular stimuli into intracellular responses [6]. Accumulating evidence has demonstrated that numerous viruses, including CVB3, activate the p38 MAPK subfamily to facilitate inflammatory responses, apoptosis, and viral replication in infected cells [7–11]. The p38 MAPK inhibitor SB203580 suppresses CVB3-induced Hela cell apoptosis and viral progeny release, suggesting that the p38 MAPK signaling is essential for cytopathic effects and life cycle of CVB3 in infected cells [12]. Pharmacological inhibition of p38 MAPK phosphorylation has been shown to effectively suppress CVB3 replication and release both in vitro and in vivo [5, 13]. In addition, MAP2K3 is one of the direct upstream activators of p38 MAPK [14]. At 48 and 72 h post human cytomegalovirus infection, increased activity of MAP2K3 is critical to maintain p38 activation, which is necessary for viral DNA replication [15]. Therefore, targeting the MAP2K3/p38 MAPK signaling would be a promising therapeutic approach against CVB3 infection.

MicroRNAs (miRNAs) are small noncoding RNA molecules that post-transcriptionally inhibit gene expression by binding to specific sequences within the 3′ untranslated region (3′UTR) of target mRNAs [16]. Previous reports have demonstrated that many viruses encode miRNAs or exploit host miRNAs to modulate host and/or viral gene expression. For example, Kaposi’s sarcoma-associated herpesvirus can encode 12 miRNA precursors, which generate 25 mature miRNAs. Some of them share seed sequences with host miRNAs, targeting host mRNAs involved in immune responses [17, 18]. In addition, human immunodeficiency virus type 1 (HIV-1) induces miRNA-29a that specifically targets HIV-1 3′UTR in infected cells to modulate its own life cycle [19]. Based on miRNA-mediated host-virus interactions, using miRNAs to target mechanisms that promote viral infection and replication is an emerging and promising antiviral strategy. Its feasibility has been demonstrated in several studies, such as miR-130a-mediated suppression of pyruvate kinase in liver and red blood cells inhibits pyruvate production, which is critical for hepatitis B and C virus replication [20], and the mimics of miR-124, miR-24, and miR-744 that target the p38 MAPK signaling exhibit antiviral effects in both influenza and respiratory syncytial virus infection [21].

In our previous study, miRNA-21 has been found to be upregulated in heart tissues of mice with CVB3-induced myocarditis [22]. MiRNA-21 plays an important role in cardiovascular development and is dysregulated in various cardiovascular diseases [23], such as cardiac hypertrophy [24], fibrosis [25], and myocardial infarction [26]. Interestingly, MAP2K3 is a direct target of miRNA-21 and miRNA-21 mimics can effectively inhibit MAP2K3 expression in hepatocellular carcinoma [27]. Thus, we hypothesized that miRNA-21 might be involved in the MAP2K3/p38 MAPK signaling during CVB3 infection.

In the present study, we found that miRNA-21 may exert a protective role against CVB3 infection in Hela cells and mouse model through targeting the MAP2K3/P38 MAPK signaling pathway. These findings may enrich our understanding of miRNA-21 function and the underlying mechanism in CVB3-induced diseases, which provides a potential therapeutic approach for the treatment of CVB3 infection.

## Materials and methods

### Virus and animals

The pCVB3M strain was a generous gift from Decheng Yang at the University of British Columbia, British Columbia, Canada, and was passaged in HeLa cells [28]. Male BALB/c mice (6 weeks old) were purchased from the Institute of Laboratory Animal Sciences of China (Beijing, China). The animal study was approved by the Review Board of the Capital Institute of Pediatrics (Beijing, China).

### miRNA detection

Cellular RNAs were extracted using the miRNeasy MiniKit (Qiagen) according to the manufacturer’s instructions and were then reverse transcribed using a TaqMan MicroRNA Reverse Transcription Kit (Life Technologies). Mature miRNA levels were detected by the TaqMan MicroRNA Assay (Life Technologies) using relative quantitative methods as described previously [28]. U6 RNA was detected as the endogenous control for data normalization. Reverse transcription primer was 5′ CTCAACTGGTGTCGTGGAGTCGGCAATTCAGTTGAGTCAACA 3′, the sense and anti-sense primers are 5′-ACACTCCAGCTGGCTAGCTTATCAGACTGATG-3′ and 5′-CTCAACTGGTGTCGTGGA-3′. All q-RT-PCR experiments were repeated in triplicates with the no-template as a control.

### Luciferase reporter assay

HEK293T cells were seeded in 6-well plates at 5 × 10^5^ per well for 24 h before transfection. Then 5 μl of 20 μM of miR-21 or mutant miR-21 has no human targets were transfected into HEK293T cells simultaneously with 250 ng of wild-type or mutant MAP2K3 3′UTR-pmirGLO plasmid using lipofectamine 2000 (Invitrogen, Carlsbad, CA) according to the manufacturer’s instructions. The luciferase activity was measured using a dual-luciferase Reporter Assay System (Promega, Madison) 24 h after transfection and the relative luciferase activity value was achieved against the renilla control.

### Virus infection in vitro

HeLa cells were grown in complete medium (DMEM supplemented with 10% newborn calf serum) to 70 to 80% confluence prior to infection. HeLa cells were then infected with CVB3 at a multiplicity of infection (MOI) of 10, unless otherwise indicated, or sham infected with phosphate-buffered saline (PBS) for 1 h in serum-free DMEM. Cells were then washed with PBS and cultured in complete medium for the indicated periods of time.

### MAP2K3 inhibition and P38 MAPK inactivation

ON-TARGET plus SMART pool MAP2K3 siRNA containing 4 siRNA against MAP2K3 was purchased from Dharmacon (M-003509-03; Lafayette, CO, USA), and was transfected into HeLa cells using Lipofectamine 2000 at a final concentration of 20 nM according to the manufacturer’s instructions. siRNA unrelated to the human genome was also used as a control. The medium was replaced after 12 h, CVB3 infection were carried out 24 h later at an MOI = 1.

SB203580, P-P38 MAPK inhibitor were purchased from MedChem Express. HeLa cells were treated with 50 μM SB203580 for 30 min and then infected with CVB3 at MOI = 1.

#### Lentivirus generation and in vitro and in vivo infection

To generate lentiviral vectors overexpressing miRNA-21, oligonucleotides of miRNA-21 forward (5′-GAGGATCCCCGGGTACCGGTTTATCAAATCCTGCCTGAC-3′) and reverse primer (5′-CACACATTCCACAGGCTAGACCAGACAGAAGGACCAG-3′) were synthesized, based on the sequence of human miRNA-21 (5′-uagcuuaucagacugauguuga-3′, MIMAT0000076) from the miRBase database, and the oligonucleotides were introduced into pGCSIL-GFP plasmid (GeneChem Co. Ltd. Shanghai, China). To construct lentivirus, pHelper 1.0 and pHelper 2.0 were purchased from Shanghai GeneChem Co. Ltd. Generation of lentivirus and viral titration evaluation were conducted as described previously [29]. Lentivirus containing miRNA-21 sequence was designated as Lenti-miRNA-21. Lentivirus containing a miRNA sequence that has no identified target in mammalian cells was also constructed as control and was designated as Lenti-CON.

For HeLa cells, Lenti-miRNA21 was transduced at MOI = 10, CVB3 infection were carried out 72 h later at an MOI = 1.

For susceptible mice, 2 × 10^8^ transduction units Lenti-miRNA-21 were intravenously injected into each mouse via the caudal vein. Mice were then inoculated intraperitoneally with a dose of 1 × 10^5^ plaque forming units (PFU, LD50) of CVB3 virus per mice. Some mice were monitored daily for the onset of paralysis and survival. Other mice were euthanized on days 3, 5 and 7 after infection with CVB3 (n = 10 per group). Experiments were carried out 3 times.

For miRNA-21 expression in the heart of mice, excised mouse heart was weighed and homogenated, total RNA was obtained from homogenated cells with TRIzol reagent and following procedures were as forementioned.

### Caspase-3 activity assay

Caspase-3 activity assay was measured according to the manufacturer’s suggestion (R&D Systems). HeLa cells were transduced with Lenti-miRNA-21 at an MOI = 10 followed by CVB3 infection at an MOI = 1 72 h later. 6 and 12 h post infection, cell lysates were harvested and subjected to caspase-3 activity assay by using of a fluorogenic substrate.

### Cell viability assays

Cell viability was measured using an MTS assay kit (Promega, Madison) according to the manufacturer’s instructions. HeLa cells were transduced with Lenti-miRNA-21 and infected with CVB3 as above and were then incubated with MTS solution for 2 h at the indicated time point, and the absorbance was measured at 492 nm using a microplate reader. The survival value for the absorbance measured from the control cells was defined as 100%, and the values obtained from the other infected cells were converted to a ratio based on the value of the control sample.

### Viral plaque assay and histological analysis in heart tissues

To detected viral titers, supernatants from HeLa cells were collected at 6 h, 12 h and 24 h post infection, and cell debris was discarded by centrifugation for 5 min at 2000 rpm. To detect viral titers within HELA cells, the lysates of infected cells were collected as previously described [30]. Briefly, cells were trypsinized at 12 h postinfection and counted, followed by PBS rinses and centrifugation at 400×*g* for 5 min. Cell pellets were resuspended in 1 ml of DMEM containing 5% fetal calf serum and subjected to three freeze–thaw cycles using liquid nitrogen and a heating block set to 37 °C. Samples were then centrifuged at 10,000×*g* for 10 min at 4 °C to remove cell debris. To detect the viral loads of tissues, hearts from mice were weighed, homogenized in 0.5 ml MEM, and centrifuged at 1000 rpm for 10 min. The viral titers in the supernatants, cell lysates, and infected tissues were analyzed by viral plaque assay as previously described [28], and were expressed as PFU/ml, /5 × 10^5^ cells, and/gram, respectively. To assess the severity of myocarditis, paraffin-embedded sections of heart tissues were stained with hematoxylin–eosin and examined histopathologically for evidence of inflammation and necrosis [28]. To assess apoptosis of cardiomyocyte, Tunel assay and immunohistochemistry staining for cleaved caspase 3 were performed (Tunel Apoptosis assay kit, Beyotime Technology, China, anti-cleaved caspase 3 monoclonal antibody, Cell Signaling Technology, Inc, China) following the manufacture’s instruction or described previously [29].

### Annexin V-FITC/PI staining and flow cytometry

Cells were pooled, pelleted by centrifugation, washed once with ice-cold PBS, and resuspended in binding buffer (10 mM HEPES–NaOH [pH 7.4], 140 mM NaCl, 2.5 mM CaCl_2_) to a concentration of 10^6^/ml. 0.1 ml of cell suspension was transferred to a 5-ml tube and incubated with 5 μl of Annexin-V and 5 μl of PI for 15 min at 25 °C in the dark. Samples were analyzed by flow cytometry within 1 h on a FACScan flow cytometer (BD Biosciences). Results are presented as the percentage of early apoptotic (Annexin-V^+^ PI^−^) and late apoptotic (Annexin-V^+^ PI^+^).

### Protein detection

Hela cells or homogenated mice heart tissue were collected at the indicated time point and were lysed in RIPA buffer (Kangwei, Beijing, China). Antibodies for detecting MAP2K3, P38 MAPK, P-P38 MAPK, HSP 27, P-HSP 27, cleaved caspase-3, Bax and GAPDH were purchased from Cell Signaling Technology (Cell Signaling Technology, Inc, China). Antibodies for detecting viral VP1 was purchased from Leica Biosystems Newcastle. Western blotting was conducted as described previously [31].

### Statistical analysis

All statistical analyses were performed using the SPSS 13.0 computer software program (SPSS, Inc., Chicago, IL). Survival was analyzed using the log-rank (Mantel–Cox) method. The significance of variability among the experimental groups was determined by the Mann–Whitney U test. All differences were considered statistically significant at *p *< 0.05.

## Results

### CVB3 infection in HeLa cells activates miR-21 expression, which targets the MAP2K3 3′UTR

**Fig. 1:**
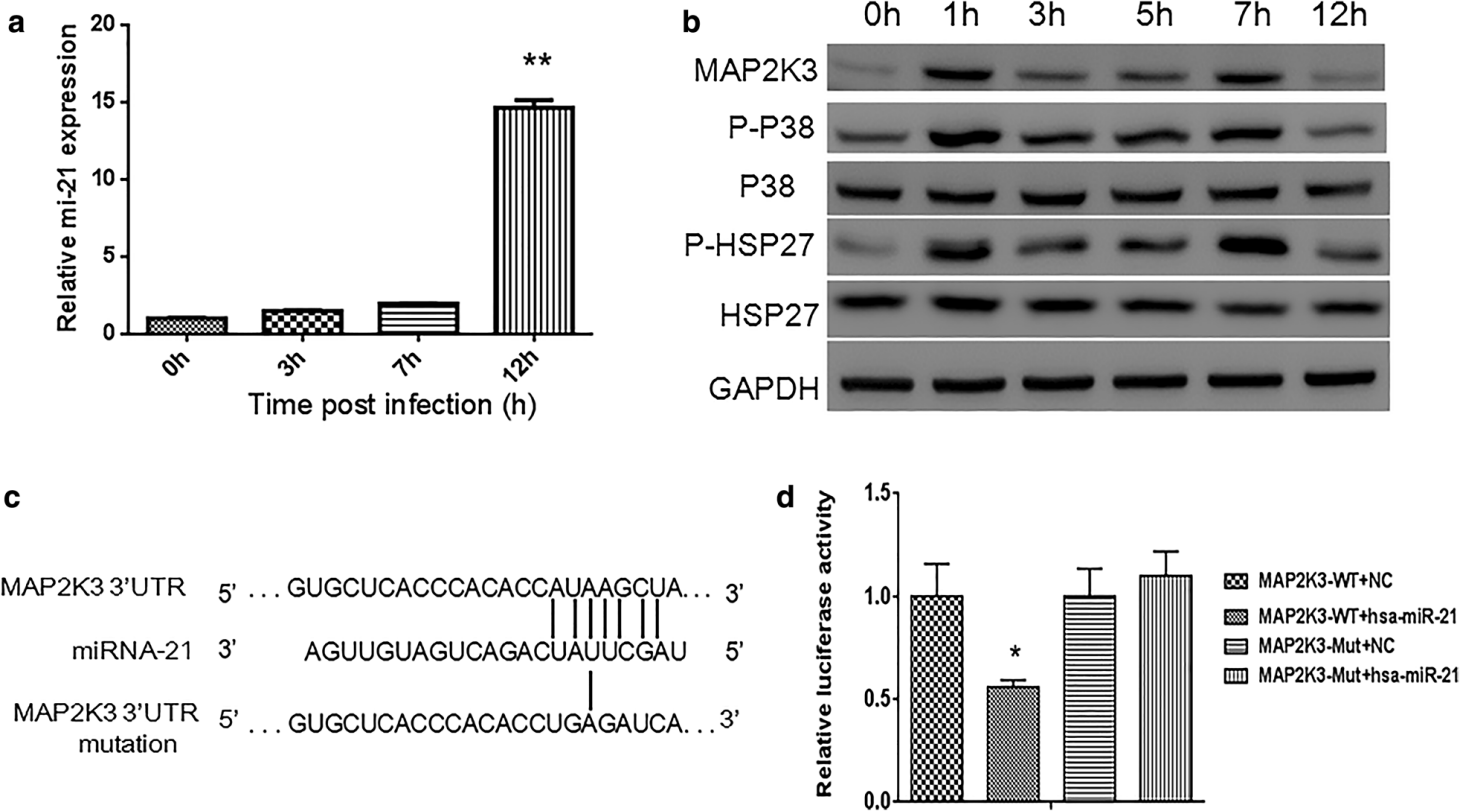
CVB3 infection activates P38 MAPK pathway and upregulates miRNA-21 expression, which targets the 3′UTR of MAP2K3. HELA cells were infected with CVB3 at MOI = 10 and profiling of cellular miRNA-21 and P38 MAPK proteins expression were detected, function of miRNA-21 in targeting MAP2K3 were also validated. **a** miRNA-21 expression was detected by Real-time PCR in CVB3-infected HeLa cell at indicated time post infection, MOI = 10. All data were normalized to U6 RNA. **b** Time courses for CVB3 stimulation and phosphorylation of MAP2K3 and proteins in the P38 pathways in HeLa cells. GAPDH is shown as a loading control. Shown were representative results of 3 independent tests. **c** Sequence alignment of miRNA-21 with putative binding sites within the wild-type and mutant 3′-untranslated regions of MAP2K3. **d** Relative luciferase activity of HeLa cells transfected with wild-type or mutant MAP2K3 3′UTR luciferase reporter with miRNA-21 or a mutant miRNA-21. Results represent the means and standard deviations from three independent triplicated experiments (N = 6), (*p < 0.05)

In our previous study, we performed expression profiling of cellular miRNAs in the heart of CVB3-infected mice and determined that miRNA-21 is overexpressed in the acute stage [22]. We infected HeLa cells with CVB3 at MOI = 10 and assessed miRNA-21 levels post infection. Real-time PCR results confirmed that miRNA-21 was increased after CVB3 infection, with approximately almost 15-fold at 12 h (Fig. 1a). CVB3 infection in HeLa cells leads to activation of P38 MAPK, which is downstream of MAP2K3 [12, 13]. To determine whether CVB3 induced miRNA-21 upregulation could affect MAP2K3 levels, we examined continuous changes of MAP2K3 expression upon CVB3 infection. MAP2K3 was upregulated significantly 1 h post CVB infection and sustained at high levels 7 h post infection, however, the expression level dropped at 12 h (Fig. 1b), which seems to be the result of high level of miRNA-21 and in line with our previous in vivo result [22]. To further assess the effects of CVB3 on associated P38MAPK pathways, we examined phosphorylation of P38 MAPK (P-P38) and phosphorylation of heat shock protein 27 (P-HSP27), which is a downstream substrate of p38 MAPK pathway and found the expression of P-P38 and P-HSP27 were almost in accordance with MAP2K3, both of them had high levels at 2–7 h while decreased at 12 h (Fig. 1b).

Bioinformatics analysis suggests that the 3′UTR of MAP2K3 is a candidate target for miRNA-21 (Fig. 1c). To assess this possibility, we performed luciferase reporter assays in HEK293 cells. miRNA-21 mimic specifically reduced the activity of a reporter containing the 3′UTR of MAP2K3, while no effect was apparent with the control miRNA or mutated target (Fig. 1d). On the basis of these findings, we conclude that MAP2K3 participate in CVB3 infection process and miRNA-21 has the potential to inhibit MAP2K3 expression.

### P38 MAPK participates in CVB3 infection process and MAP2K3 Inhibition reduces phosphorylation of P38 MAPK and CVB3 release

**Fig. 2:**
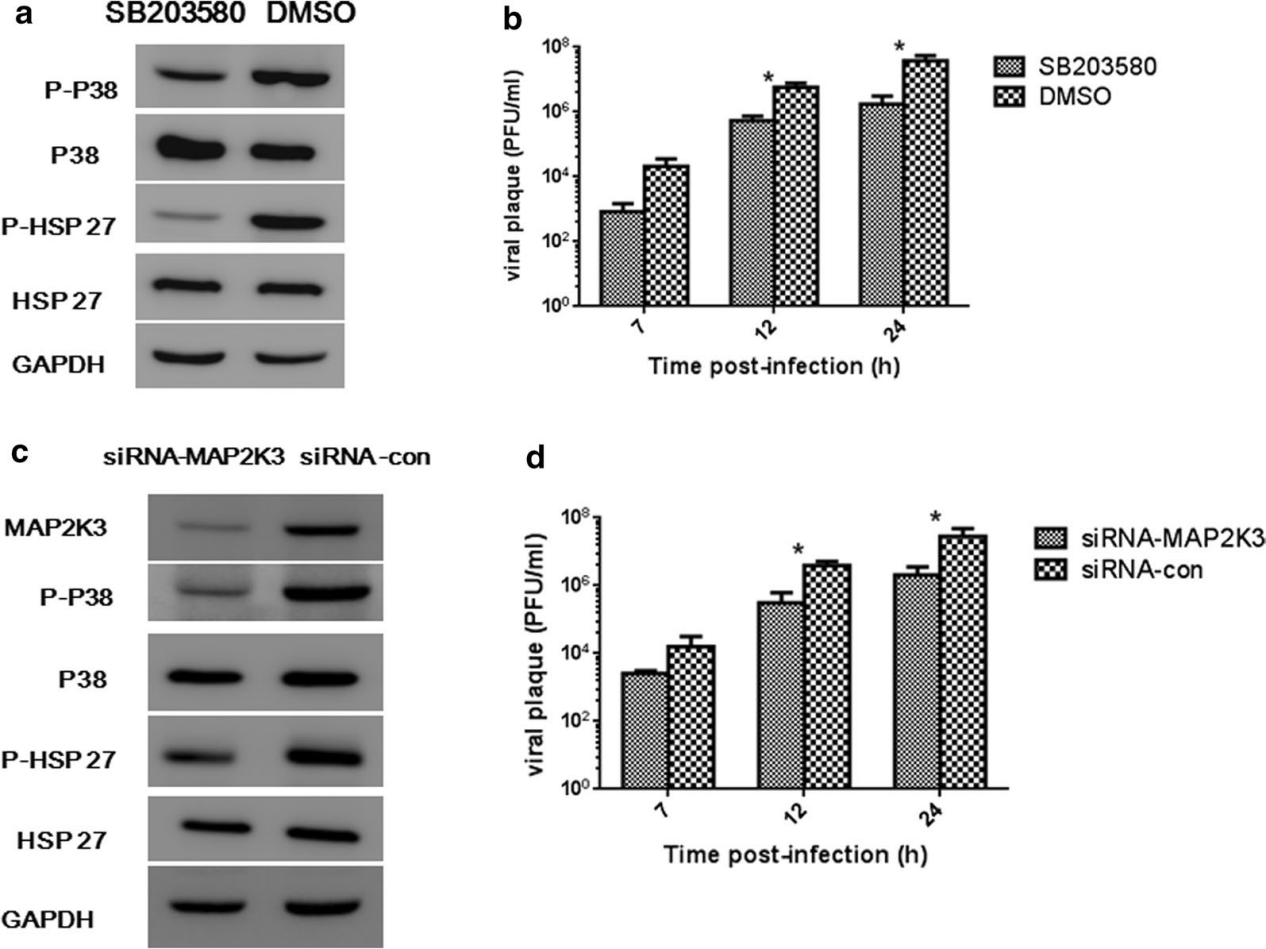
Inactivation of P38 MAPK and reduction of MAP2K3 could reduce CVB3 replication. Hela cells were pretreated with P38 inhibitor SB203580 or MAP2K3 specific siRNA (MAP2K3-siRNA as shown in the figure) and were then infected with CVB3 at MOI = 1. DMSO and siRNA has no targets (siRNA-con as shown in the figure) were used as control. **a** Western bolt was conducted to detect P-P38 and downstream P-HSP27 in the presence of SB203580 7 h post CVB3 infection, DMSO was loaded as control group. **b** CVB3 replication in the supernatants of SB203580 treated cells at indicated time point were detected. **c** Western bolt was conducted to detect MAP2K3, P-P38 and downstream P-HSP27 expression in MAP2K3 specific siRNA treated cells 7 h post CVB3 infection. siRNA has non targets to the human genome was tested as a control. Shown were representative results from 3 independent tests. **d** CVB3 replication in the supernatants of siRNA-MAP2K3 treated cells were detected at indicated time point. Virus titer values represent the means and standard deviations of three independent experiments, (*p < 0.05)

CVB3 infection could activate P38 MAPK signaling pathway, we further investigated the roles of P38 MAPK in viral progeny release. P38 MAPK specific inhibitor SB203580 was used in our experiment. HeLa cells were treated with 50 μM of SB203580 for 30 min, and P-P38 MAPK and the downstream P-HSP27 were reduced (Fig. 2a). We further detected CVB3 in the supernatant and found that inhibition of P38 activation induced significant reduction of viral progeny release (Fig. 2b).

To evaluate the role of MAP2K3 in CVB3-infected HeLa cells, we used siRNA to specifically knock down MAP2K3 and CVB3 were infected at MOI = 1. 24 h later when MAP2K3 was inhibited, MAP2K3 and P-P38 MAPK expression were evaluated at 7 h post CVB3 infection, when they were commonly at peak expression as in Fig. 1b. We found their expression were attenuated in MAP2K3-siRNA-expressing cells (Fig. 2c). Furthermore, CVB3 titers were decreased in the supernatant of MAP2K3-siRNA treated cells at 12 h and 24 h (Fig. 2d). These results suggest that P-P38 MAPK participates in the CVB3 replication process, and inhibition of MAP2K3 could reduce P-P38 MAPK expression and CVB3 viral progeny release.

### Exogenous miRNA-21 inhibits CVB3 release by targeting MAP2K3 in HeLa cells

**Fig. 3:**
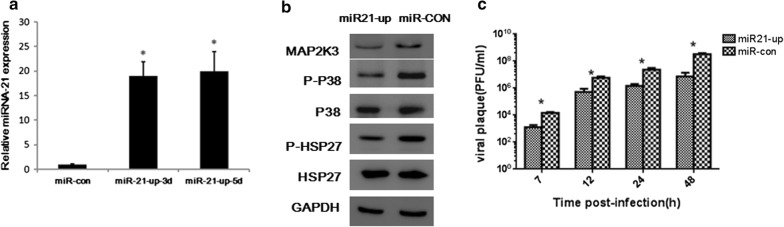
Exogenous miRNA-21 inhibits CVB3 replication by targeting MAP2K3 in HeLa cells. HeLa cell were transduced with Lenti-miRNA-21 at MOI = 10, and were then infected with CVB3 at MOI = 1 3 days later (miR21-up as shown in the figure). Cells transduced with Lenti-CON followed by CVB3 infection were used as control group (miR-con as shown in the figure). **a** miRNA-21 expression in HeLa cells after transduction with Lenti-miRNA-21 or control lentivirus 3 days and 5 days later, miR-con represents cells transduced with Lenti-CON and miR21-up represents cells transduced with Lenti-miRNA-21. **b** Western blotting was conducted to detect MAP2K3, P-P38 MAPK and P-HSP27 expression in HeLa cells 7 h post CVB3 infection in Lenti-miRNA-21 transduced cells. GAPDH was loaded as a control group. Shown were representative results from 3 independent test. **c** CVB3 replication in the supernatants post CVB3 infection at indicated time point in Lenti-miRNA-21 transduced cells. Virus titer values represent the means and standard deviations of three independent experiments, (*p < 0.05)

Given its high expression in CVB3 infection process and ability to target MAP2K3, we reasoned that expression of exogenous miRNA-21 might provide an approach for ameliorating the effects of CVB3 infection. To investigate this possibility, we constructed lentivirus containing the miRNA-21 sequence (designated as Lenti-miRNA-21) and lentivirus containing miRNA with no identified target in mammalian cells (designated as Lenti-CON). Hela cells were infected with CVB3 at MOI = 1 3 days post transduction of lentivirus, when miRNA-21 expression was upregulated (Fig. 3a). As expected, MAP2K3, P-P38 MAPK, and P-HSP27 levels were reduced in Lenti-miRNA-21 group compared with the Lenti-CON group 7 h post CVB3 infection, while total P38 MAPK and HSP27 levels were not changed (Fig. 3b). To evaluate whether exogenous miRNA-21 can restrain CVB3 release in HeLa cells, we collected the supernatant and performed viral plaque assays, and found that viral load was significantly decreased post infection (Fig. 3c).

### MiRNA-21 does not affect CVB3 replication while reversing CVB3-induced caspase-3 activation in host cells in early phases of infection

**Fig. 4:**
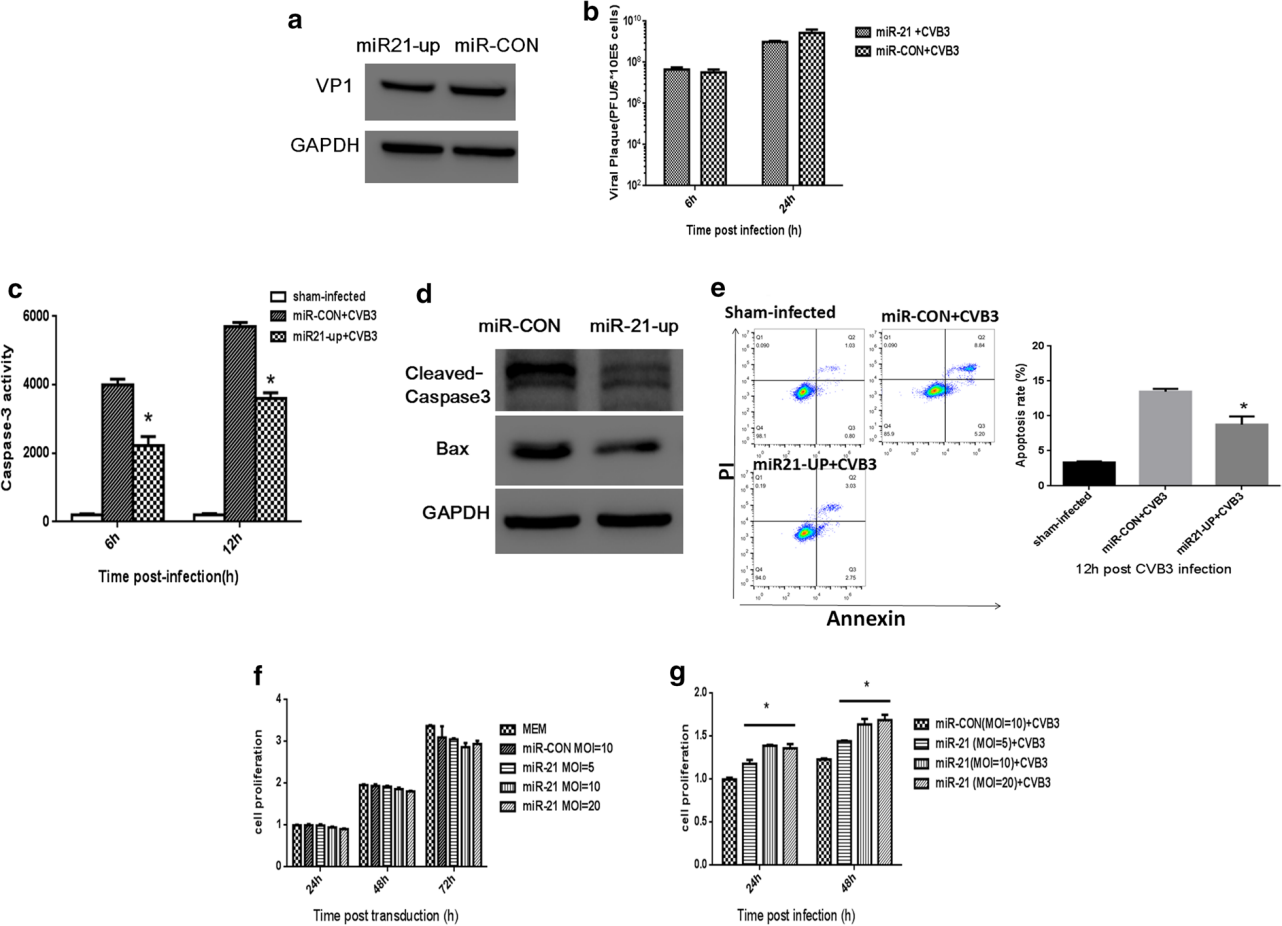
The effect of miRNA-21 on viral replication and host cell apoptosis and proliferation. HeLa cells were transduced with Lenti-miRNA-21 at MOI = 10, followed by CVB3 infection at MOI = 1 at 3 days after transduction. Cells transduced with Lenti-miR-CON and sham-infected cells were used as controls. **a** Western blot assay was conducted to detect CVB3 capsid protein VP1 at 12 h post infection. Representative results from 3 independent experiments are shown. **b** Intracellular viral titers were detected at 12 h post CVB3 infection. **c** Cell lysates were harvested and caspase-3 activity was detected using a fluorogenic substrate at indicated time points. **d** Immunoblot analysis of whole-cell protein lysates from CVB3 infected cells probed with cleave-caspase 3 and Bax antibody. **e** CVB3 induces apoptosis was measured by Annexin-V binding to externalized phosphatidylserine, representative FACS data of annexin-V^+^ apoptotic cells were shown. Cell viability assay was performed at multiple time points, with different amounts of miRNA-21 without (**f**) or with CVB3 infection **(g)**. The viability of HeLa cell was determined by MTS assay at indicated time points, (*p < 0.05)

CVB3 infection and replication in cells may cause cytopathic effects and eventually cell death, and the last step of life cycle of an enterovirus is to release viral progeny into the medium [32]. To investigate whether miRNA21-inhibited CVB3 progeny release was due to suppressed CVB3 replication, we detected the expression of CVB3 capsid protein VP1 in infected Hela cells at 6 h following infection. As shown in Fig. 4a, VP1 protein levels were comparable between miRNA-21 and control groups. Consistently, no significant change was observed between the viral loads within miRNA-21- and miRNA-CON-transfected HELA cells at 6 h and 24 h post infection (Fig. 4b). These results suggest that viral replication in the cells was not affected by miRNA-21 at least within 24 h post infection.

Considering that decreased viral progeny release may result from inhibited host cell apoptosis and the p38 MAPK activation is linked to apoptotic signals [33, 34], we next sought to investigate the effect of miRNA-21 on apoptosis post CVB3 infection. As shown in Fig. 4c, CVB3 infection caused a dramatic increase in the caspase-3 activity in miR-CON-transfected cells compared with sham infection in a time-dependent manner, suggesting that CVB3 might induce apoptosis in infected cells. Importantly, miRNA-21 could significantly reverse the inductive effect of CVB3 on caspase-3 activation compared with miR-CON. Apoptosis related Cleaved-caspase 3 and Bax expression 12 h post infection were also detected and were found decreased in the miRNA-21 treated group, we further assayed apoptosis rate by Annexin V-FITC/PI staining assay and found miRNA-21 could inhibit apoptosis rate compared with control-group, Fig. 4d, e. These results suggested a potential protective role of miRNA-21 against CVB3-induced apoptosis in infected cells. Indeed, miRNA-21 showed no effect on the proliferation of uninfected Hela cells while significantly promoting the proliferation in CVB3-infected cells in a dose- and time-dependent manner (Fig. 4f, g), indicating that miRNA-21 may protect infected cells against CVB3-induced apoptosis, which further improves cell survival. Taken together, these results suggest that the inhibition of CVB3 release from miRNA-21-overexpressing cells is likely attributed to miRNA-21-exerted protection against CVB3-induced apoptosis at least in the early phases of infection.

### miRNA-21 overexpression inhibits CVB3 pathogenesis in mice

**Fig. 5:**
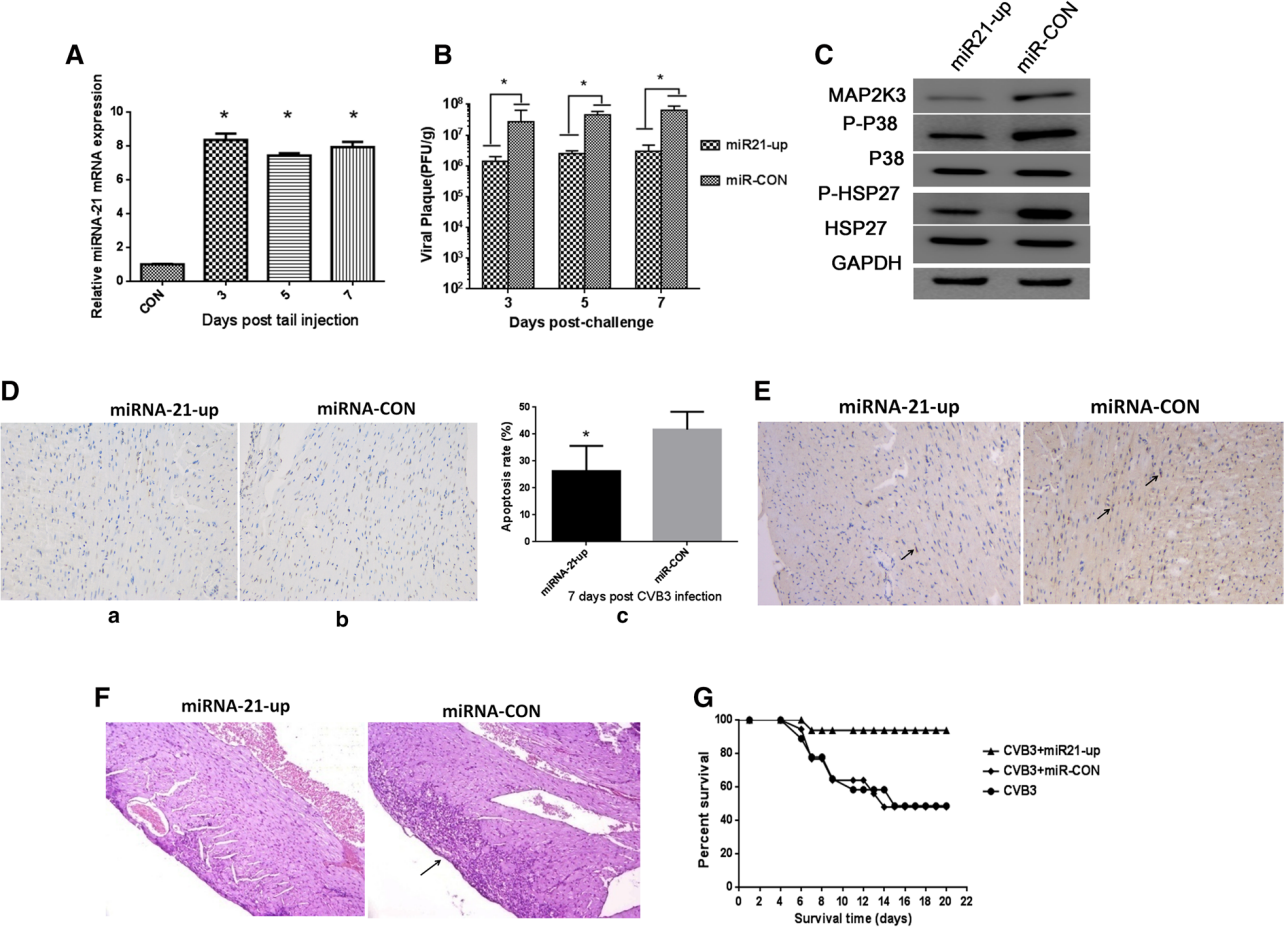
miRNA-21 overexpression inhibits CVB3 pathogenesis in mice. To detect whether high levels of miRNA-21 have effect on CVB3 infected mice, 2 × 10^8^ transduction units of lenti-miRNA-21 were intravenously injected into mice via the caudal vein. Each mice were then inoculated intraperitoneally with a dose of 1 × 10^5^ plaque forming units (PFU, LD50) of CVB3 virus (miR21-up as shown in the figure). Mice transduced with Lenti-CON followed by CVB3 infection were used as control group (miR-con as shown in the figure). **A** Lenti-miRNA-21 were intravenously injected into mice and miRNA-21 levels were increased significantly in the heart. **B** CVB3 viral titers detection were conducted at indicated time point in the hearts of mice after treatment with Lenti-miRNA-21 (n = 5), and viral titers were decreased compared with control group. **C** Western blot of MAP2K3, P-P38 MAPK, and P-HSP 27 expression in the heart of mice transduced with Lenti-miRNA-21. GAPDH was loaded as control group. Shown were representative results from 3 independent tests. **D** TUNEL assay were performed at day 7 post infection and percentage of TUNEL positive cells in each sample were shown, representative images were shown, original magnification, ×200. **E** Immunohistochemistry for cleaved-caspase 3 at day 7 post infection were detected, representative immunohistochemistry observation of each group was shown, examples of cleaved-caspase 3 expression are shown (black arrow), original magnification, ×200. **F** Histological analysis in the heart tissues at day 7 post infection of mice pretreated with Lenti-miRNA-21 or control. Examples of necrosis, calcification are shown (black arrow). Original magnification, ×200. **G** Evaluation of the antiviral effects of Lenti-miRNA-21 on mice survival rate. Mice were pretreated with 2 × 10^8^ transduction units of lentivirus (miR21-up and miR-con as shown in the figure) or MEM (CVB3 as shown in the figure) as indicated followed by infection of LD50 CVB3 and survival was evaluated, (*p < 0.05)

Based on the in vitro experiments, we hypothesized that miRNA-21 may also alleviate CVB3 pathogenesis in a mouse model of viral myocarditis. To evaluate this possibility, we injected 2 × 10^8^ transduction units of lenti-miRNA-21 via the caudal veins of mice. miRNA-21 expression was confirmed to be dramatically upregulated in the heart at 3 to7 days post injection (Fig. 5A). 1 × 10^5^ pfu (LD50) of CVB3 were intraperitoneally inoculated into mice at day 3, and CVB3 titers were found decreased in the lenti-miRNA-21 group as compared to the control group (Fig. 5B), which confirms that exogenous miRNA-21 can inhibit viral replication. MAP2K3 was also decreased in heart tissues from miRNA-21-expressing mice, which is consistent with our in vitro results. Furthermore, P-P38 MAPK and P-HSP27 were decreased in the miRNA-21-expressing mice, which is consistent with a role for MAP2K3 in activating the P38 MAPK pathway (Fig. 5C).

To further explore whether miRNA-21 could inhibit apoptosis in vivo, Tunel assay reflecting severity of myocyte apoptosis and immunohistochemistry staining for cleaved-caspase 3 expression 7 days post infection were performed, compared with control group, percentage of TUNEL positive cells were decreased in miRNA-21 treated group (Fig. 5D), and cleaved-caspase 3 were also down-regulated, which indicates the function of miRNA-21 in the inhibition of apoptosis process in CVB3 infected mice (Fig. 5E).

To determine the physiological outcome of miRNA-21 overexpression in CVB3-infected mice, we examined the hearts of infected mice. Cardiac histopathology revealed that mice with high expression of miRNA-21 showed only minor dropsy and hemorrhage, while mice in the control group showed significant necrosis and signs of mononuclear cell infiltration on day 7 post infection (Fig. 5F). Furthermore, the survive rate was clearly improved in miRNA-21-expressing mice after CVB3 infection (Fig. 5G), which is consistent with a role for miRNA-21 in attenuating CVB3 infection in the heart.

## Discussion

Activation of the MAP2K3/P38 MAPK signaling pathway is essential for CVB3 infection [5, 12, 13]. Our previous study showed that miRNA-21 that potentially targets MAP2K3 is upregulated in the heart of mice with CVB3-induced myocarditis [22]. In addition, miRNA-21 has been shown to have beneficial effects on myocardial infarct size in murine models [26]. However, the association of miRNA-21 with the MAP2K3/P38 MAPK signaling in the context of CVB3 infection remains unclear. In the present study, we found that miRNA-21 may inhibit CVB3 viral progeny release through targeting the MAP2K3/P38 MAPK signaling pathway in vitro. In addition, miRNA-21 pretreatment could reduce viral titer within the heart and improve pathological alterations and clinical outcomes in a mouse model of CVB3 infection. These findings suggest that miRNA-21 may serve as a potential therapeutic agent in the treatment of CVB3-associated diseases.

In this study, we detected dynamic expression of miRNA-21 and the MAP2K3/P38 MAPK signaling in CVB3-infected HeLa cell line which is commonly used in the studies of CVB3 [31]. When compared with those observed at earlier time points, the miRNA-21 expression in Hela cells was dramatically upregulated at 12 h following CVB3 infection, whereas the protein levels of MAP2K3 and phosphorylated downstream targets, including P-P38 and P-HSP27, were remarkably decreased accordingly, suggesting an association between miRNA-21 and the MAP2K3/P38 MAPK signaling in the context of CVB3 infection. The results of a luciferase reporter assay further confirmed that MAP2K3 is a potential target of miRNA-21, which is consistent with a previous report [27]. These findings indicate that CVB3 infection can induce miRNA-21 upregulation in host cells, leading to a suppression of the MAP2K3/P38 MAPK signaling.

Following the intracellular replicative process, the virus is released to complete the final stage of the viral life cycle [32]. Significant reduction in the amount of released virus in response to siMAP2K3 or P38 inhibitor was observed at 12 and 24 h postinfection, suggesting that activation of the MAP2K3/P38 MAPK signaling is indispensable for CVB3 life cycle. Similar results were also observed in infected cells overexpressing miRNA-21 compared with control miRNA first at 7 h post infection and persisted for at least 48 h, accompanied by downregulation of MAP2K3 and dephosphorylation of downstream targets. These findings suggest that miRNA-21 may inhibit CVB3 progeny release via suppression of the MAP2K3/P38 MAPK signaling, which is supported by previous studies [13, 35]. The inhibition ability of miRNA-21 was comparable to siRNA-MAP2K3 and P38 MAPK specific inhibitor SB203580, and could reach to an extent for more than tenfold, which was improtant in the viral controlling process.

To further investigate whether the inhibitory effect of miRNA-21 on progeny release was due to suppressed CVB3 replication, we detected viral capsid protein VP1 expression as well as the viral load within infected Hela cells [36, 37]. No significant change in VP1 expression and viral load was observed between infected cells overexpressing miRNA-21 and control miRNA, suggesting that miRNA-21 might not affect CVB3 replication in infected cells at least within 24 h post infection. Because decreased viral progeny release may result from inhibited host cell apoptosis [33], we speculated that miRNA-21 might protect infected cells from apoptosis. Therefore, we next evaluated the caspase-3 activity and cell proliferation in miRNA-21-overexpressing infected cells. We found that miRNA-21 significantly inhibited the inducive effect of CVB3 on caspase-3 activation and promoted infected cell proliferation in a dose- and time-dependent manner. We also detected apoptosis related cleaved-caspase 3 and Bax expression in miRNA-21-treated infected cells, and apoptosis were also evaluated by Annexin-V/PI staining by flow cytometric, results showed that apoptosis related protein were decreased in the miRNA-21-treated group and apoptosis rate were also declined, suggesting that miRNA-21 might protect host cells from CVB3-induced apoptosis, leading to an improvement in cell survival. A previous study showed that overexpression of antiapoptotic proteins or treatment with a general caspase inhibitor prevents CVB3 virus progeny release at 10 h following infection, indicating that host cell apoptosis facilitates progeny release from infected cells [33]. Thus, the inhibitory effect of miRNA-21 on host cell apoptosis might contribute to decreased viral release at least during the initial stage of viral infection.

It is well-established that miRNA-21 is closely associated with the pathogenesis of cardiovascular diseases due to its abundance in cardiovascular system and frequent alteration during the development of the diseases [24–26]. However, whether miRNA-21 is favorable or unfavorable to cardiovascular system remains controversial [24, 26, 38], which may partially due to different mouse strains used in these studies. In the present study, we established a CVB3 infection model using male BALB/c mice and observed significantly reduced CVB3 titer and suppressed MAP2K3/P38 MAPK signaling in the heart tissue of miRNA-21-pretreated mice, which is in agreement with our in vitro data. In addition, the mice pretreated with miRNA-21 exhibited improved pathological alterations, decreased percentage of apoptosis and apoptosis related cleaved-caspase 3 expression in the heart, what is important, miRNA-21 treatment also prolonged survival time. These data suggest that miRNA-21 may be beneficial for the host during CVB3 infection. However, due to insufficient funding support, we did not include a miRNA-21 knockout mouse model in this study to provide more convincing evidence, which needs to be addressed in the future.

In conclusion, in the present study, we revealed a protective role of miRNA-21 against CVB3 infection through targeting the MAP2K3/P38 MAPK signaling pathway both in vitro and in vivo, and viral inhibition were attributed to the inhibition of cell apoptosis process. Our results provides miRNA-21 as a potential therapeutic agent for prevention and treatment of CVB3-induced diseases.

## Data Availability

All data generated or analyzed during this study are included either in this article.

## Abbreviations

CVB3: coxsackievirus group B type3
miRNA: microRNA
MAPK: mitogen-activated protein kinase
HSP27: heat shock protein
MOI: multiplicity of infection
siRNA: small interference RNA
UTR: untranslated regions
VP1: viral protein 1

## References

[1] Liu PP, Mason JW (2001). Advances in the understanding of myocarditis. Circulation.

[2] He F, Yao H, Xiao Z, Han J, Zou J, Liu Z (2014). Inhibition of IL-2 inducible T-cell kinase alleviates T-cell activation and murine myocardial inflammation associated with CVB3 infection. Mol Immunol.

[3] Schultz JC, Hilliard AA, Cooper LT, Rihal CS (2009). Diagnosis and treatment of viral myocarditis. Mayo Clin Proc.

[4] Esfandiarei M, McManus BM (2008). Molecular biology and pathogenesis of viral myocarditis. Annu Rev Pathol.

[5] Dai Q, Zhang D, Yu H, Xie W, Xin R, Wang L, Xu X, He X, Xiong J, Sheng H (2017). Berberine restricts coxsackievirus B type 3 replication via inhibition of c-jun N-terminal kinase (JNK) and p38 MAPK activation in vitro. Med Sci Monit.

[6] Thouverey C, Caverzasio J (2015). Focus on the p38 MAPK signaling pathway in bone development and maintenance. Bonekey Rep..

[7] Gong J, Shen XH, Chen C, Qiu H, Yang RG (2011). Down-regulation of HIV-1 infection by inhibition of the MAPK signaling pathway. Virol Sin..

[8] Steer SA, Moran JM, Christmann BS, Maggi LB, Corbett JA (2006). Role of MAPK in the regulation of double-stranded RNA- and encephalomyocarditis virus-induced cyclooxygenase-2 expression by macrophages. J Immunol..

[9] Spaziani A, Alisi A, Sanna D, Balsano C (2006). Role of p38 MAPK and RNA-dependent protein kinase (PKR) in hepatitis C virus core-dependent nuclear delocalization of cyclin B1. J Biol Chem.

[10] Gillis PA, Okagaki LH, Rice SA (2009). Herpes simplex virus type 1 ICP27 induces p38 mitogen-activated protein kinase signaling and apoptosis in HeLa cells. J Virol.

[11] Mizutani T, Fukushi S, Saijo M, Kurane I, Morikawa S (2005). JNK and PI3 k/Akt signaling pathways are required for establishing persistent SARS-CoV infection in Vero E6 cells. Biochim Biophys Acta.

[12] Si X, Luo H, Morgan A, Zhang J, Wong J, Yuan J, Esfandiarei M, Gao G, Cheung C, McManus BM (2005). Stress-activated protein kinases are involved in coxsackievirus B3 viral progeny release. J Virol.

[13] Marchant D, Dou Y, Luo H, Garmaroudi FS, McDonough JE, Si X, Walker E, Luo Z, Arner A, Hegele RG (2009). Bosentan enhances viral load via endothelin-1 receptor type-A-mediated p38 mitogen-activated protein kinase activation while improving cardiac function during coxsackievirus-induced myocarditis. Circ Res.

[14] Derijard B, Raingeaud J, Barrett T, Wu IH, Han J, Ulevitch RJ, Davis RJ (1995). Independent human MAP-kinase signal transduction pathways defined by MEK and MKK isoforms. Science.

[15] Johnson RA, Huong SM, Huang ES (2000). Activation of the mitogen-activated protein kinase p38 by human cytomegalovirus infection through two distinct pathways: a novel mechanism for activation of p38. J Virol.

[16] Hammond SM (2015). An overview of microRNAs. Adv Drug Deliv Rev.

[17] Skalsky RL, Cullen BR (2010). Viruses, microRNAs, and host interactions. Annu Rev Microbiol.

[18] Zhu Y, Haecker I, Yang Y, Gao SJ, Renne R (2013). gamma-Herpesvirus-encoded miRNAs and their roles in viral biology and pathogenesis. Curr Opin Virol..

[19] Nathans R, Chu CY, Serquina AK, Lu CC, Cao H, Rana TM (2009). Cellular microRNA and P bodies modulate host-HIV-1 interactions. Mol Cell.

[20] Duan X, Li S, Holmes JA, Tu Z, Li Y, Cai D, Liu X, Li W, Yang C, Jiao B (2018). MicroRNA 130a regulates both hepatitis C virus and hepatitis B virus replication through a central metabolic pathway. J Virol..

[21] McCaskill JL, Ressel S, Alber A, Redford J, Power UF, Schwarze J, Dutia BM, Buck AH (2017). Broad-spectrum inhibition of respiratory virus infection by microRNA mimics targeting p38 MAPK signaling. Mol Ther Nucleic Acids..

[22] Zhang Q, Xiao Z, He F, Zou J, Wu S, Liu Z (2013). MicroRNAs regulate the pathogenesis of CVB3-induced viral myocarditis. Intervirology.

[23] Cheng Y, Zhang C (2010). MicroRNA-21 in cardiovascular disease. J Cardiovasc Transl Res..

[24] Sayed D, Rane S, Lypowy J, He M, Chen IY, Vashistha H, Yan L, Malhotra A, Vatner D, Abdellatif M (2008). MicroRNA-21 targets Sprouty2 and promotes cellular outgrowths. Mol Biol Cell.

[25] Yuan J, Chen H, Ge D, Xu Y, Xu H, Yang Y, Gu M, Zhou Y, Zhu J, Ge T (2017). Mir-21 promotes cardiac fibrosis after myocardial infarction via targeting Smad7. Cell Physiol Biochem.

[26] Dong S, Cheng Y, Yang J, Li J, Liu X, Wang X, Wang D, Krall TJ, Delphin ES, Zhang C (2009). MicroRNA expression signature and the role of microRNA-21 in the early phase of acute myocardial infarction. J Biol Chem.

[27] Xu G, Zhang Y, Wei J, Jia W, Ge Z, Zhang Z, Liu X (2013). MicroRNA-21 promotes hepatocellular carcinoma HepG2 cell proliferation through repression of mitogen-activated protein kinase-kinase 3. BMC Cancer..

[28] He F, Yao H, Wang J, Xiao Z, Xin L, Liu Z, Ma X, Sun J, Jin Q, Liu Z (2015). Coxsackievirus B3 engineered to contain microRNA targets for muscle-specific microRNAs displays attenuated cardiotropic virulence in mice. J Virol.

[29] Yao H, Zhang Y, He F, Wang C, Xiao Z, Zou J, Wang F, Liu Z (2012). Short hairpin RNA targeting 2B gene of coxsackievirus B3 exhibits potential antiviral effects both in vitro and in vivo. BMC Infect Dis.

[30] Steinmann E, Doerrbecker J, Friesland M, Riebesehl N, Ginkel C, Hillung J, Gentzsch J, Lauber C, Brown R, Frentzen A (2013). Characterization of hepatitis C virus intra- and intergenotypic chimeras reveals a role of the glycoproteins in virus envelopment. J Virol.

[31] Xin L, Ma X, Xiao Z, Yao H, Liu Z (2015). Coxsackievirus B3 induces autophagy in HeLa cells via the AMPK/MEK/ERK and Ras/Raf/MEK/ERK signaling pathways. Infect Genet Evol..

[32] Carthy CM, Granville DJ, Watson KA, Anderson DR, Wilson JE, Yang D, Hunt DW, McManus BM (1998). Caspase activation and specific cleavage of substrates after coxsackievirus B3-induced cytopathic effect in HeLa cells. J Virol.

[33] Carthy CM, Yanagawa B, Luo H, Granville DJ, Yang D, Cheung P, Cheung C, Esfandiarei M, Rudin CM, Thompson CB (2003). Bcl-2 and Bcl-xL overexpression inhibits cytochrome c release, activation of multiple caspases, and virus release following coxsackievirus B3 infection. Virology.

[34] Wada T, Penninger JM (2004). Mitogen-activated protein kinases in apoptosis regulation. Oncogene.

[35] Pin AL, Houle F, Guillonneau M, Paquet ER, Simard MJ, Huot J (2012). miR-20a represses endothelial cell migration by targeting MKK3 and inhibiting p38 MAP kinase activation in response to VEGF. Angiogenesis.

[36] Li X, Xia Y, Huang S, Liu F, Ying Y, Xu Q, Liu X, Jin G, Papasian CJ, Chen J (2015). Identification of the interaction of VP1 with GM130 which may implicate in the pathogenesis of CVB3-induced acute pancreatitis. Sci Rep..

[37] Li M, Su Y, Yu Y, Yu Y, Wang X, Zou Y, Ge J, Chen R (2016). Dual roles of calpain in facilitating Coxsackievirus B3 replication and prompting inflammation in acute myocarditis. Int J Cardiol.

[38] Ye X, Zhang HM, Qiu Y, Hanson PJ, Hemida MG, Wei W, Hoodless PA, Chu F, Yang D (2014). Coxsackievirus-induced miR-21 disrupts cardiomyocyte interactions via the downregulation of intercalated disk components. PLoS Pathog.

